# A Computationally Efficient and Causal Frequency Domain Formalism for Hemodynamics Allowing for Nonlinearities and Generalized Coupling Conditions

**DOI:** 10.1002/cnm.70104

**Published:** 2025-10-07

**Authors:** Mikael Karlsson, Mina Nashed, Tamer Elnady, Mats Åbom

**Affiliations:** ^1^ Engineering Mechanics KTH Royal Institute of Technology Stockholm Sweden; ^2^ Faculty of Engineering Ain Shams University Cairo Egypt

**Keywords:** frequency domain, hemodynamics, pulse wave propagation, reduced order in silico models

## Abstract

Reduced order hemodynamic models are an increasingly important complementary tool to in vivo measurements. They enable effective creation of large datasets with well‐defined parameter variations, which can be used, for example, for training machine learning models, conducting virtual studies of intervention strategies, or for the development of pulse wave analysis algorithms. Here, a 1D frequency domain formalism for pulse wave propagation in the cardiovascular system is presented. Using the scattering matrix formulation, a computationally efficient and causal solution is obtained, including possible source terms and nonideal coupling conditions. Local nonlinear effects, as those seen in stenoses or aneurysms, are introduced via an iterative procedure, achieving as good accuracy as state‐of‐the‐art time‐domain solvers while being significantly more computationally efficient. The new formalism has been successfully validated against well‐documented reference cases from the literature.

## Graphical abstract

A 1D frequency domain formalism for pulse wave propagation is presented and validated against established benchmarks. It is computationally efficient, causal and enables general nonideal coupling conditions. Nonlinear effects, as seen locally in, for example, stenoses, are introduced via an iterative procedure.

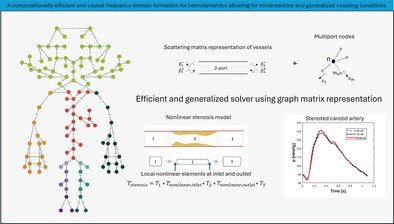

## 1 Introduction

The arterial pulse has long been recognized as an indicator of cardiovascular status, with its diagnostic utility dating back to ancient times. Historically, it was believed to be “sent everywhere simultaneously” [1]. The concept of the arterial pulse as a wave propagation phenomenon began to be explored in the 18th century by Leonard Euler, who formulated the fundamental system of nonlinear conservation equations. Subsequently, the development of accurate constitutive relations (“tube law”) occurred predominantly during the nineteenth and early twentieth centuries (refer to Parker [1] for a comprehensive historical account). The relationship between wave speed (phase velocity) and vessel elasticity not only facilitates wave propagation analysis but also holds intrinsic clinical significance. Wave speed has emerged as an independent biomarker of cardiovascular disease (CVD) [2]. While age‐related variations are natural [3], deviations can signal CVD. Typically, studies focus not on local phase velocity but on the average—often termed pulse wave velocity (PWV)—between two accessible points.

The actual local arterial pressure at a given monitoring point results from the superposition of forward and backward traveling waves. Reflections may occur due to changes in the geometry or properties of the vessel, such as bifurcations, anomalies, or localized stiffening of the vessel wall. Consequently, analysis of the morphology of the pulse wave can yield extensive information about a patient's cardiovascular status. Historically, these measurements required specialized equipment and trained personnel, but recent developments of readily available noninvasive sensors have revolutionized this field, enabling large‐scale in vivo research studies and even the integration of such measurements into routine health monitoring devices, including smartwatches.

However, clinical trials still remain costly, time‐consuming, and suffer from measurement uncertainty. Perhaps the most significant limitation is the lack of reliable control data, as well‐defined parameter variations are difficult to achieve in vivo. An invaluable complementary tool is in silico modeling, which spans from fully resolved three‐dimensional (3D) fluid–structure interaction models to simplified lumped element approaches [4]. For pulse wave propagation studies, low‐order one‐dimensional (1D) models have proven effective in capturing the essential features of pulse waves relevant for physiological analysis. A comprehensive review of the diverse 1D models available can be found in Alastruey et al. [5]. These models enable the creation of large virtual patient cohorts with precisely defined parameter variations at a low computational cost. Such virtual cohorts have been extensively utilized to evaluate algorithms [6, 7, 8], establish reference databases [9, 10], test machine learning algorithms for anomaly detection [11, 12, 13, 14], and deepen the understanding of various pathologies [15, 16, 17, 18].

From a clinical and health monitoring perspective, a significant application lies in personalized models, often referred to as digital twins [19, 20]. These models enable patient‐specific diagnosis and monitoring of CVDs, facilitating tasks such as the identification of vascular anomalies (e.g., rupture risk of aneurysms or stenosis progression), surgical intervention planning [21], and heart monitoring. The vascular model is informed by sensor data from a limited number of monitoring points, which are used to optimize the model parameters either offline or in real‐time. A considerable challenge arises from the substantial interpatient parameter variability, even among healthy individuals (see a meta‐study on typical parameter ranges in Charlton et al. [9]). Uncertainty analysis serves as a valuable tool for identifying critical parameters relevant to specific applications [22]. However, even with parameter reduction—such as the identification of 16 influential parameters out of 73 in Huberts et al. [22]—the degrees of freedom in the optimization problem remain considerable. This underscores the need for developing computationally efficient methods.

In this paper, we present a formalism for efficiently setting up and solving a 1D representation of the cardiovascular system. The nonlinear Navier–Stokes (NS) equations can be solved in the time domain. However, by neglecting the effects of convective acceleration, the NS equations become linear, enabling the derivation of frequency‐domain solutions that are more effectively solved using Fourier analysis. This approach, broadly referred to as Womersley's oscillatory theory [23], will be further explored in the theory section. With a linear relationship between pressure and velocity, the arterial tree can be modeled using transmission line analogies from electric circuit theory, where individual vascular elements are represented by complex‐valued impedances [24]. This modeling technique is particularly suited for cascade (serial‐coupled) networks and can yield either backward [25] or forward [26] transmission lines, depending on the implementation. We introduce a general “multiport” formalism for solving a low‐order network model in the frequency domain, which accommodates arbitrary coupling conditions at nodes and sources at any position. This formalism has been extensively developed and applied to industrial applications [27, 28] of plane wave propagation in ducts with mean flow. Among its advantages is its computational efficiency, which has been demonstrated to be optimal for complex network systems [29]. The computational effort required naturally depends on the complexity of the model. Some models encompass the entire circulatory system, including the heart, pulmonary components, and arterial and venous structures [30], making them useful for system‐level studies. For specific applications, the complexity of the model can often be significantly reduced while maintaining acceptable accuracy [31].

We begin by introducing the methodology, followed by the multiport representations of vessels, junctions, anomalies, vascular beds, and heart boundary conditions. Observations are provided regarding the assumptions made and the challenges in estimating the required input parameters. Although this is a frequency‐domain model, it is demonstrated that local nonlinearities, such as those associated with stenoses, can be incorporated. The models are subsequently validated against benchmark cases from the literature, examples ranging from single vessels to full aorta models.

## 2 Theory

### 2.1 Generalized Low Order Network

Multiport networks are a standard approach for analyzing branched waveguide problems in engineering and various other fields. A multiport is a “black box” that establishes a causal relationship between a set of input (**x**) and output (**y**) state variables, which are typically assumed to have the same dimensions (*N*). Here, *N* represents the number of degrees of freedom of the *N*‐port. For a linear and time‐invariant system, the most general frequency‐domain representation of a multiport can be expressed as [32]:

(1) $$\hat{y}=G\hat{x}+{\hat{y}}^{s}$$

where the circumflex denotes a Fourier transform $\left[∼\exp\left(-\mathrm{i\omega t}\right)\right]$, G represents the [*NxN*] matrix for passive properties, ${\hat{y}}^{s}$ accounts for active properties or sources within the multiport, and ω and *t* denote angular frequency and time, respectively. Depending on the choice of state variables, various formulations are possible. In arterial hemodynamics, 1D frequency domain or Fourier methods are commonly referred to as impedance lines. This approach typically involves two‐ports (*N* = 2), where arterial pressure (*p*) and volume flow (*q*) serve as state variables and having an admittance/impedance formulation relating the input and output of the waveguide (vessel). Standard coupling conditions assume continuity in pressure and conservation of volume flow across connection points (“nodes”). The proposed multiport methodology can generalize these coupling conditions. Impedance line methods often proceed from a known peripheral boundary condition, working backward (“backward algorithm” [25]) toward the heart, specifically to the load impedance on the aortic valve. Alternatively, the approach can originate from a known heart source, moving forward [26].

As highlighted by Flores et al. [33], convective nonlinear effects are generally weak in normal vascular systems but can become significant at localized defects, such as stenoses or aneurysms. Consequently, pulse propagation is predominantly linear, with nonlinearities treated as localized phenomena. This strategy is used in other fields utilizing linear waveguide models, such as intake or exhaust systems in internal combustion engines [34].

For complex networks with arbitrary node coupling conditions, Eversman [35] proposed that the most computationally efficient approach is the scattering‐matrix formulation. Assuming 1D, or plane waves, multiports are defined in terms of forward (${\hat{p}}_{+}$) and backward (${\hat{p}}_{-}$) traveling pressure wave amplitudes. This formalism inherently reflects the fundamental waveguide nature of the problem, where the corresponding time domain 1D wave equation has two solutions, propagating to the left and right, respectively. Hence, being derived from the causal time domain solution the scattering formulation, unlike admittance/impedance formulations, ensures that causality is preserved. Causality is crucial for accurate coupling to the time domain, particularly when integrating a nonlinear heart model. Furthermore, scattering‐matrix formulations provide an intuitive interpretation of multiport behavior in terms of reflection (*R*) and transmission coefficients (*T*). As demonstrated by Glav and Åbom [29], they also enable a straightforward incorporation of active elements, whether these sources exist within the element itself or at the nodes.

**FIGURE 1:**
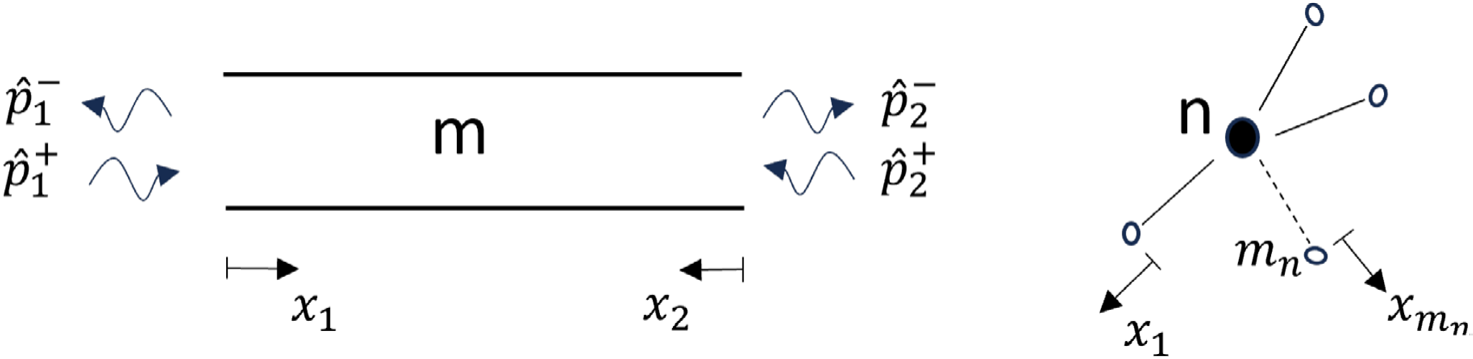
Definitions of positive directions of a two‐port element (“vesssel”) *m* and a multiport node *n* connected to $m_{n}$ number of elements. Plane waves are assumed. The node positive direction is chosen to be consistent with the connected two‐ports.

For an active two‐port *m* from interface 1 to 2, as defined in Figure 1, the scattering‐matrix formulation is:

(2) $${\left[\begin{array}{c} {\hat{p}}_{1-}\\ {\hat{p}}_{2-} \end{array}\right]}_{m}={\left[\begin{array}{cc} R_{1} & T_{21}\\ T_{12} & R_{2} \end{array}\right]}_{m}{\left[\begin{array}{c} {\hat{p}}_{1+}\\ {\hat{p}}_{2+} \end{array}\right]}_{m}+{\left[\begin{array}{c} {\hat{p}}_{1-}^{s}\\ {\hat{p}}_{2-}^{s} \end{array}\right]}_{m}$$

where *R* and *T* denote reflection and transmission coefficients, respectively, and the superscript *s* denotes a source. The junction or connection between elements, referred to as a “node,” can be modeled either as a multiport element or simply as a point (0D‐element). The latter case is the standard assumption, implying continuity of pressure and volume flow across the node. In later sections, we will explore how general multiport models for nodes can be derived, using a bifurcation as a representative example. Glav and Åbom [29] have proposed a general formalism for two‐port networks, extending the earlier work of Eversman [35]. In this framework, all elements are represented using Equation (2), which employs [2 × 2] scattering matrices $S_{m}$ and source vectors ${\hat{p}}_{-,m}^{s}$. Nodes connecting two‐port elements are described as multiports, with the order determined by the number of two‐ports connected to a node. Both two‐port elements and nodes can be active, meaning they may include sources. As demonstrated by Glav and Åbom [29], a network that includes *M* two‐ports can be expressed as a network equation for the *2 M* unknown two‐port wave amplitudes ${\hat{p}}_{+}$:

(3) $$A{\hat{p}}_{+}=B{\hat{p}}_{-}^{s}+{\hat{q}}^{s}$$

where **A**, **B** are [*2Mx2M*] matrices assembled from the two‐port and node scattering matrices, ${\hat{p}}_{-}^{s}$ is the complete two‐port source vector and ${\hat{q}}^{s}$ is a vector containing any volume flow sources (1‐ports) at the nodes. Both source terms are [*2Mx1*] column vectors. Assuming instead a standard coupling condition at the nodes, it is possible to formulate the problem only using nodal pressures [36, 37]. It can be shown that this simplification leads to the minimum number of equations for a network [29].

It is now instructive to examine Equation (3). On the left‐hand side, the equation represents the unknown system response, ${\hat{p}}_{+}$, while the right‐hand side describes the excitation introduced by one‐ and two‐port sources. The **A** and **B** matrices are constructed exclusively from passive data. The matrix **A** characterizes how ${\hat{p}}_{+}$ is scattered throughout the network, while **B** quantifies the influence of the two‐port sources, ${\hat{p}}_{-}^{s}$, on the network's response. For a given network, Equation (3) is solved to determine all the incident wave amplitudes, ${\hat{p}}_{+}$. Once these amplitudes are obtained, the outgoing amplitudes, ${\hat{p}}_{-}$, can be derived using the two‐port equations (Equation (2)). In Glav and Åbom [29], an algorithm for assembling the **A** and **B** matrices is described, demonstrating that for large complex networks, this approach is significantly more efficient than the method proposed by Eversman [35]. The algorithm is not based on any assumption of network coupling (cascade, tree, etc.), instead the network is described as a “graph object” allowing for any degree of complexity.

Based on the algorithm proposed by Glav and Åbom [29] a code “BEATLAB” has been developed to model arterial networks. The remainder of this section presents the details of the elements utilized in the code, including nonlinear models addressing local defects such as stenoses or aneurysms. For now, sources are incorporated solely as one‐port elements at nodes; this is, for example, how the heart is represented. Future investigations may extend this framework to include two‐port sources, which could model phenomena such as localized turbulence at a stenosis or controlled external excitation of the vascular system [38].

### 2.2 Womersley Model

An advantage of using a frequency‐domain model is the ability to incorporate Womersley's [23] model for oscillatory 1‐D viscous flow. This solution provides a frequency‐dependent flow profile and volume flow, assuming a 1‐D pressure gradient along a uniform vessel. Unlike 1‐D time‐domain models, which rely on approximate flow profiles, this approach offers a more accurate representation of oscillatory flow dynamics [39]. Building on Womersley's work [23], Huo and Kassab [40] derived a 1‐D wave equation for harmonic waves propagating along the *x*‐axis of a straight, uniform vessel:

(4) $$\frac{\partial^{2}\hat{p}}{\partial x^{2}}+\kappa^{2}\hat{p}=0$$

with $\hat{p}\left(\omega ,x\right)={\hat{p}}_{\pm }\left(\omega \right)\exp\left(\mp \mathrm{i\kappa x}\right)$ and ${\hat{p}}_{\pm }=\pm Z_{0}{\hat{q}}_{\pm }$ for a free wave in ± *x* where $\hat{q}$ is the volume flow and $c_{0}$, κ, and $Z_{0}$ is the wave speed, wave number, and characteristic impedance respectively given by

(5) $$c_{0}=\sqrt{\frac{\mathrm{Eh}}{2\left(1-\upsilon^{2}\right)\rho_{0}R}}$$

(6) $$\kappa =\frac{\omega }{c_{0}\sqrt{1-F}}$$

(7) $$Z_{0}=\frac{\rho_{0}c_{0}}{\pi R^{2}\sqrt{1-F}}$$

(8) $$\alpha =R\sqrt{\frac{\omega }{\nu }}$$

(9) $$F=\frac{2J_{1}\left(\alpha i^{3/2}\right)}{\alpha i^{3/2}J_{0}\left(\alpha i^{3/2}\right)}$$

where *E* represents Young's modulus and υ Poisson's ratio of a thin elastic wall with thickness *h* of a vessel with radius *R* conveying a fluid of density $\rho_{0}$. It is typically assumed that an arterial wall is incompressible, implying a Poisson's ratio of 0.5. Finally, α refers to the Womersley number, ν is the kinematic viscosity, and $J_{0}$ and $J_{1}$ are Bessel functions of the 0th and 1st order, respectively. For vascular flow applications, the steady (0 Hz) component is important, necessitating that limit values are taken for Equations (6) and (7). By allowing the Womersley number to be complex, fluid properties can be adjusted between Newtonian and Maxwellian [33]. Frequency domain models also simplify the incorporation of vessel wall losses by treating the elastic modulus as complex valued and frequency dependent [41]. The radius in these models should correspond to the time‐averaged radius, $R_{0}$. Additionally, in the formula for wave speed (Equation (5)), the radius should be selected as $R_{d}^{2}/R_{0}$, where $R_{d}$ is the diastolic radius [33]. If the diastolic pressure is assumed to represent a stress‐free state, the time‐averaged radius can be computed as:

(10) $$R_{0}=R_{d}+\left({\hat{p}}_{0}-p_{d}\right)/K$$

where ${\hat{p}}_{0}$ represents the time‐averaged (steady) or 0 Hz pressure component, $p_{d}$ is the diastolic pressure, and $K=\mathrm{Eh}/\left(\left(1-\upsilon^{2}\right)R_{d}^{2}\right)$. In practice, the difference between the diastolic radius and the time‐averaged radius is typically only a few percent. As a result, frequency‐domain models often assume a fixed radius equal to the diastolic value for simplicity. However, by employing Equation (10) and estimating the diastolic pressure, an iterative procedure can be established to compute the time‐averaged radius for a given excitation signal.

### 2.3 Elements in the Model

Here, the basic two‐port and one‐port elements utilized in the frequency‐domain model are presented. Additionally, a method for incorporating nonlinear effects in frequency‐domain models is proposed.

#### 2.3.1 Vessel With Constant Properties

Using 1‐D free wave solutions, it is straightforward to demonstrate that the scattering matrix **S** for a straight, uniform vessel segment of length *L*, with the positive directions defined as in Figure 1, is:

(11) $$S=\left[\begin{array}{cc} 0 & \exp\left(-\mathrm{i\kappa L}\right)\\ \exp\left(-\mathrm{i\kappa L}\right) & 0 \end{array}\right]$$

This element includes transmission only with no reflection. The corresponding *backward* (2–1) transfer matrix is given as:

(12) $$T=\left[\begin{array}{cc} \cos\left(\mathrm{\kappa L}\right) & iZ_{0}\sin\left(\mathrm{\kappa L}\right)\\ \text{isin}\left(\mathrm{\kappa L}\right)/Z_{0} & \cos\left(\mathrm{\kappa L}\right) \end{array}\right]$$

Here, the same positive direction (1–2) is assumed at both the inlet and outlet. Published element models are often expressed in transfer matrix form, but it is straightforward to derive the scattering matrix using simple matrix transformations (see Appendix A). In the steady flow or 0 Hz limit, the Womersley model simplifies Equation (12) to the Hagen–Poiseuille laminar flow case:

(13) $$T=\left[\begin{array}{cc} 1 & 8\mathrm{\mu L}/\pi R^{4}\\ 0 & 1 \end{array}\right]$$

where μ is the dynamic viscosity.

For elements connected in cascade or series, the overall element properties can be efficiently computed by multiplying the transfer matrices sequentially from the inlet to the outlet [34].

#### 2.3.2 Vessels With Varying Properties

The properties, such as radius and Young's modulus, can vary along the centerline of a vessel. Assuming smooth variation, such vessels can be modeled by approximating the actual vessel as a series of N straight segments with locally constant properties [42]. The transfer matrix $T_{\mathrm{vp}}$ for the entire vessel can be estimated as:

(14) $$T_{\mathrm{vp}}=T_{1}\cdot T_{2}\cdot \ldots \cdot T_{N}$$

where $T_{n}$ (*n* = 1, 2,…, *N*) are matrices corresponding to straight vessel segments, as defined by Equations (12) and (13). The length of each segment ΔL can remain uniform. As the number of segments (*N*) increases, $T_{\mathrm{vp}}$ converges to the transfer matrix of the actual vessel. For practical applications, it suffices to ensure that ΔL is less than approximately 1/5 of the wavelength at the highest frequency of interest. Additionally, Papadakis [43] proposed that for tapered sections, the wave speed $c_{0}$ should be adjusted by a factor of $\cos\left(\theta \right)/\cos\left(\theta /2\right)$, where θ represents the tangent angle.

#### 2.3.3 Vessels With Defects

**FIGURE 2:**
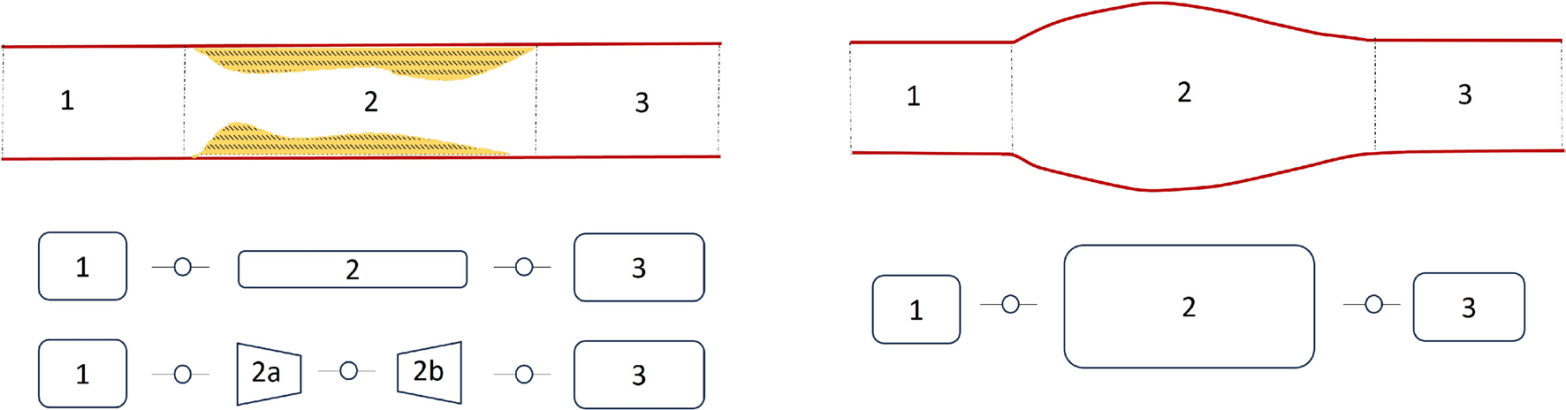
Top: Schematic of stenosis (left) and aneurysm (right) and segmentation. Bottom: Corresponding two‐port models. The second alternative for the stenosis according to Papadakis et al. [44].

Vascular defects, such as stenoses and aneurysms, can, despite often exhibiting complex 3D geometries, be represented as localized area changes in 1D vascular networks. The vessel is divided into three segments (see Figure 2), with the surrounding segments (1 and 3) maintaining the properties of the original vessel. In the defect—segment 2—the wave propagation is mainly affected by “sudden area changes + flow separation.” Flow separation occurs when smooth flow is disrupted and detaches from a surface, creating regions of recirculation, eddies, or turbulence. This leads to increased drag, energy losses, and unsteady flow behavior. In the case of stenosis, flow separation typically occurs at the inlet and outlet, where the vessel's cross‐sectional area changes abruptly. The severity of this effect increases with increasing area ratio, indicating a stronger nonlinear impact. As an alternative to modeling stenosis as a simple abrupt area change, Papadakis et al. [44] proposed a conical reduction and expansion. Such conical sections can also be modeled using the procedure described in Section 2.3.2. Additionally, a combination of these two stenosis models is possible. To account for flow separation, a nonlinear element is introduced. Assuming incompressible flow, the losses at a sudden area change or local constriction can be modeled as a lumped nonlinear two‐port element [34].

(15) $$T_{\mathrm{nl}}=\left[\begin{array}{cc} 1 & R_{\mathrm{nl}}\left({\hat{q}}_{0}\right)\\ 0 & 1 \end{array}\right]$$

where $R_{\mathrm{nl}}\left({\hat{q}}_{0}\right)$ is a nonlinear resistance that depends on the steady (0 Hz) flow component. This two‐port can now be used to add flow losses at the inlet/outlet of element 2 in Figure 2. A complete model for a stenosis or aneurysm can then be obtained by multiplying five transfer matrices

(16) $$T_{\mathrm{def}}=T_{1}\cdot T_{\mathrm{nl},\text{in}}\cdot T_{2}\cdot T_{\mathrm{nl},\text{out}}\cdot T_{3}$$

In time‐domain models, nonlinearity is typically introduced through a flow‐speed‐dependent viscosity term [45]. Here, the flow losses are instead introduced locally based on the assumption of quasi‐stationary flow and the Borda–Carnot formula for losses at a sudden area change [46]. This approach assumes that the losses—or drop in stagnation pressure—are proportional to the square of the instantaneous volume flow, $q{\left(t\right)}^{2}$. If the volume flow is represented as the sum of a steady component, $q_{0}$, and harmonics, $q_{1}\left(t\right),q_{2}\left(t\right),\ldots$, it can be expressed as:

(17) $$\begin{array}{c} q{\left(t\right)}^{2}=q_{0}^{2}+q_{1}^{2}+q_{2}^{2}+\ldots +2q_{0}\left(q_{1}+q_{2}+\ldots \right)\\ \;+2q_{1}\left(q_{2}+q_{3}+\ldots \right)+\ldots \end{array}$$

In regions prone to stenosis, such as the lower limbs and carotid arteries, steady flow tends to dominate. For such cases, the model can be simplified by retaining only one nonlinear term: the dominant steady flow component, $q_{0}^{2}$, along with the linear oscillating terms. Equation (17) can thus be reduced to:

(18) $$q{\left(t\right)}^{2}\approx q_{0}^{2}+2q_{0}\left(q_{1}+q_{2}+\ldots \right)$$

Assuming incompressible flow, the continuity of volume flow implies that the drop in stagnation pressure across the area jump can be expressed as $\Delta {\hat{p}}_{n}=R_{\mathrm{nl}}\left({\hat{q}}_{0}\right)\cdot {\hat{q}}_{n}$, for n=0,1,2,…. The resistance, $R_{\mathrm{nl}}$, can be estimated using the Borda–Carnot formula [46]. For a stenosis, this can be written as:

(19) $$R_{\mathrm{nl}}\left({\hat{q}}_{0}\right)\;=\frac{\delta_{n}\rho_{0}{\hat{q}}_{0,s}C_{L,\text{in}/\text{out}}}{2A_{s}^{2}}{\left(1-\sigma_{s}\right)}^{2}$$

where based on Equation (18) $\delta_{n}$ equals 1 for n=0 and 2 for n>0, $A_{s}$ is the stenosis cross‐sectional area, $C_{L,\text{in}/\text{out}}$ represents the flow loss coefficient at the stenosis inlet/outlet, and $\sigma_{s}$ denotes the stenosis area contraction ratio. Loss coefficients can be obtained from handbooks [47] or standard textbooks for ideal cases [46]. For a sudden expansion, the expected value is $C_{L,\exp}=1$, while for a sudden contraction one expects $C_{L,\mathrm{con}}=0.5$. Following the measurements by Seeley and Young [48] for a sudden “contraction + expansion” where a total $C_{L}$ value of 1.52 was found we choose $C_{L,\mathrm{con}}=0.52$. Using the Papadakis [44] model, a single nonlinear element can be introduced between the two conical sections, based on the total flow loss coefficient. Equations (15) and (19) can be readily reformulated to apply to an aneurysm, wherein the $C_{L}$ values (expansion/contraction) at the inlet/outlet should be reversed. The volume flow at the inlet/outlet required to compute the nonlinear two‐port depends solely on the steady flow 0 Hz component, ${\hat{q}}_{0,s}=q_{0,s}$. Since this represents incompressible flow, the value can be taken from any of the nodes.

Including the nonlinear elements means that an iterative solution procedure is required. Initially, the network Equation (3) is solved while neglecting nonlinear effects. The resulting volume flows are then used to estimate nonlinear losses via Equation (15), which is employed to update the network model. These updated network equations are solved, and the process is repeated until convergence is achieved. For the cases investigated thus far, the procedure has demonstrated rapid convergence; for example, the stenosis case discussed in this paper required fewer than five iterations. It can be noticed that Flores‐Gerónimo et al. [38] proposed a similar frequency domain model for nonlinear losses at a stenosis. In their model the nonlinear term is added to the viscosity and distributed along the length of the stenosis. A different value for the total $C_{L}$ (1.2) was used and the 0 Hz component was not iterated.

#### 2.3.4 One‐Port Windkessel Elements

**FIGURE 3:**
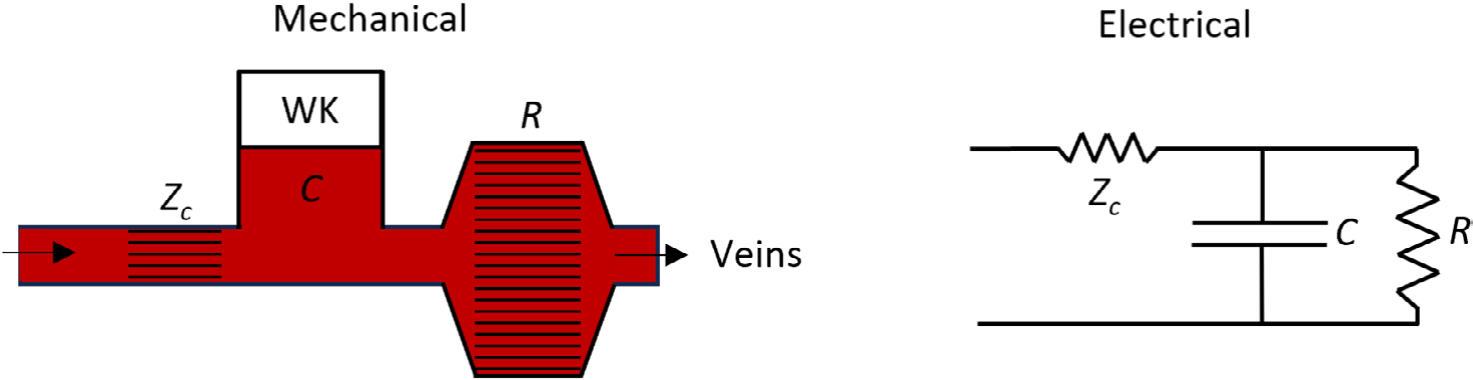
Mechanical and electrical analogy of the three‐element Windkessel (WK3) model. (Westerhof et al. [49]).

Stephen Hales (1733) proposed a simple model of the arterial tree to explain the relatively smooth arterial blood flow despite the pulsatile action of the heart. He suggested that the interaction between the heart and arteries resembled a water pump connected to an air chamber (known as “Windkessel” in German). Otto Frank (1899) later formalized this concept as a two‐element parallel impedance circuit comprising total arterial compliance (*C*) and total peripheral resistance ($R_{t}$). More recently, the Windkessel model has been extended to three‐ and four‐element configurations (WK3 and WK4) [49]. Today, the WK3 model (illustrated in Figure 3) is widely used in 1D models to define boundary conditions for vascular beds, as described by, for example, Reymond et al. [50]. In our framework, this corresponds to a passive one‐port node represented by:

(20) $${\hat{p}}_{+}=R_{\mathrm{wk}}{\hat{p}}_{-}$$

where $R_{\mathrm{wk}}=\left(Z_{\mathrm{wk}}-Z_{0}\right)/\left(Z_{\mathrm{wk}}+Z_{0}\right)$, and $Z_{\mathrm{wk}}$ is the WK3 impedance given by [50]:

(21) $$Z_{\mathrm{wk}}=Z_{c}+{\left(\mathrm{j\omega C}+1/R\right)}^{-1}$$

For peripheral vessel boundaries, Zc=ReZ0, where $Z_{0}$ is the characteristic impedance (see Equation (7)) of the terminal vessel segment preceding the WK3. Here, *C* represents the total volume compliance beyond the boundary, and $R=R_{t}-Z_{c}$, where $R_{t}$ denotes the total peripheral resistance of the relevant vascular bed. At low frequencies, the boundary impedance $Z_{\mathrm{wk}}$ approaches the total vascular bed resistance, $R_{t}$. At high frequencies, $Z_{\mathrm{wk}}$ converges to the real part of $Z_{0}$, thereby producing a reflection‐free boundary.

#### 2.3.5 One‐Port Heart Model

Following the proposed formalism, the heart is an active one‐port node

(22) $${\hat{p}}_{+}=R_{s}{\hat{p}}_{-}+{\hat{p}}_{+}^{s}$$

where $R_{s}$ is the source (“heart”) reflection coefficient. The normal ansatz is a constant volume flow source, which implies prescribing a source with $R_{s}=1$ and source impedance $Z_{s}\to \infty$. Presenting time‐domain pulse wave morphologies using realistic heart sources is a common and illustrative method in hemodynamic studies. Most validation cases available in the literature are presented in this form. For example, the Pulse Wave Database [9] provides clinicians with insights into typical pulse wave shapes at various monitoring positions as a function of age. For frequency‐domain methods, the relative spectral composition is a key issue. The harmonic energy of a typical heart signal is concentrated within the first 10 heart rate orders (see Section 3 Validation, Figure 8), meaning only a few frequency components need to be computed.

### 2.4 Bifurcations

Bifurcations play a critical role in wave propagation analysis within the vascular tree. In these regions, traveling waves are scattered due to changes in characteristic impedance even in healthy systems. However, bifurcations can also act as sites for flow separation and the development of anomalies. It is commonly assumed that vascular systems “autoregulate” to minimize losses at the bifurcation node. Under such conditions, the bifurcation can be approximated as a single nodal point, assuming continuity in pressure and volume velocity. This approximation is reasonable for a healthy system at rest. To extend this framework to bifurcations with anomalies, the previously derived stenosis and/or aneurysm elements, see Equation (15), can be incorporated, with the anomaly positioned at the end closest to the node. The steady loss coefficient, see Equation (19), needed (one for each flow path in the bifurcation) are often expressed in terms of the relative volume flow ratio between the inlet and one of the downstream branches m: $C_{L}\left(q_{\text{in}0},q_{m0}\right)$. It can be found in handbooks [47], with low cost steady state CFD (Reynolds Averaged Navier–Stokes, RANS) simulations or even lower order approximations [51]. The methodology for deriving the resulting node scattering matrix from the two‐port element scattering matrices is detailed in Appendix B. This approach will be illustrated for one of the validation cases later. While this approach compensates for changes in characteristic impedance and flow losses in the individual elements, it does not account for all interaction effects between the elements at the node.

**FIGURE 4:**
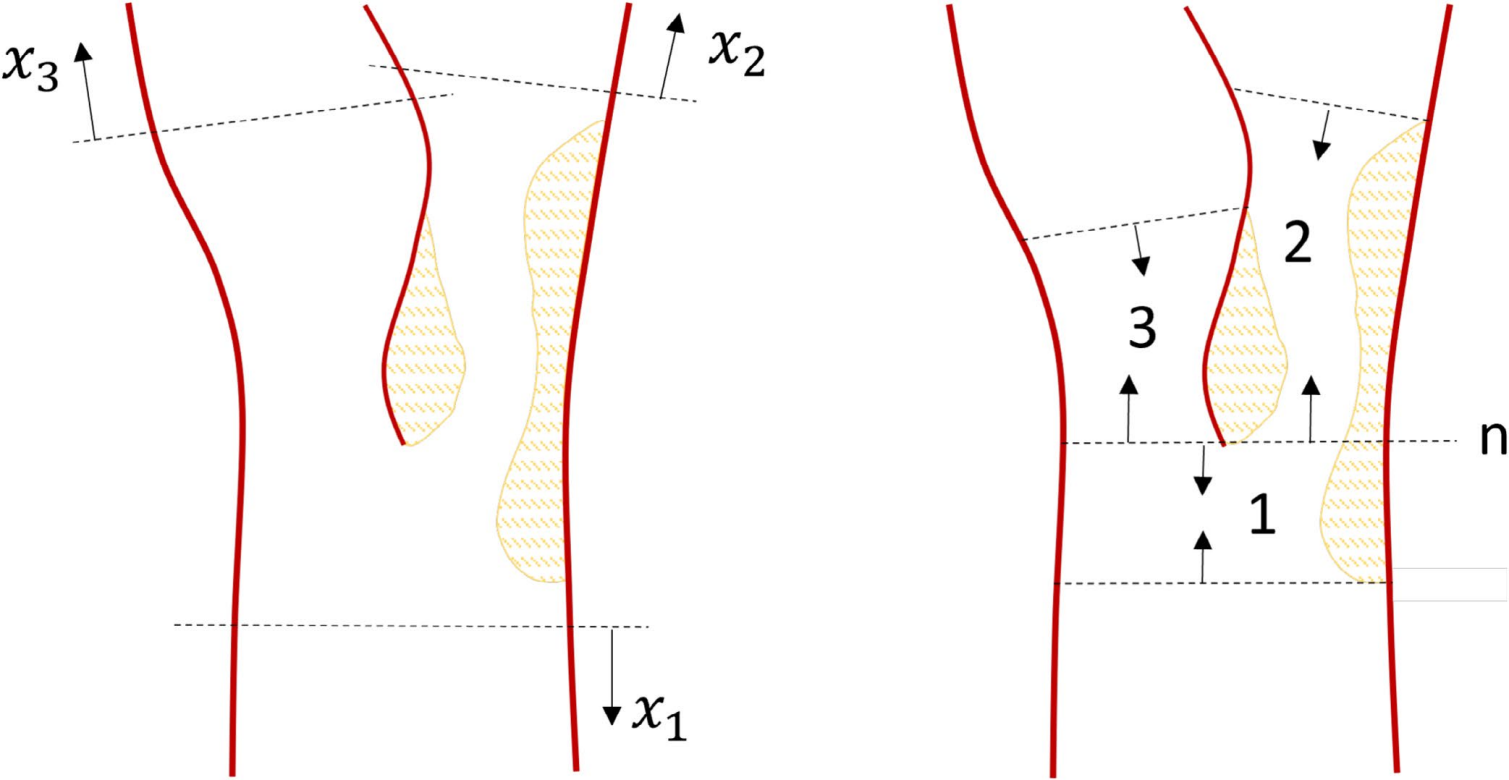
Schematic example of a carotid bifurcation with stenosis represented as a three‐port node (left) or made up of three two‐port elements connected at node n (right). Compare to the definition of nodes and two‐port elements in Figure 1.

Alternatively, treating the bifurcation directly as a multiport, as illustrated in Figure 4, these additional interaction effects can be considered. Interfaces are instead positioned outside the anomaly or flow separation region, where linearity can be assumed. Although wave propagation effects can be included, the multiport is typically compact, that is, small relative to the wavelength. The most common bifurcation involves one vessel splitting into two at a defined angle. This scenario can be modeled by extending Equation (2) to a three‐port configuration (with adjustments to the sign convention, as it is a node [29]):

(23) $${\left[\begin{array}{c} {\hat{p}}_{1+}\\ {\hat{p}}_{2+}\\ {\hat{p}}_{3+} \end{array}\right]}_{n}={\left[\begin{array}{ccc} R_{1} & T_{21} & T_{31}\\ T_{12} & R_{2} & T_{32}\\ T_{13} & T_{23} & R_{3} \end{array}\right]}_{n}{\left[\begin{array}{c} {\hat{p}}_{1-}\\ {\hat{p}}_{2-}\\ {\hat{p}}_{3-} \end{array}\right]}_{n}+{\left[\begin{array}{c} {\hat{p}}_{1+}^{s}\\ {\hat{p}}_{2+}^{s}\\ {\hat{p}}_{3+}^{s} \end{array}\right]}_{n}$$

This format aligns with the node scattering matrix derived in Appendix B from the combination of three elements. The effects of flow interaction and losses are included in the passive scattering matrix [52]. There are different methods for determining this node scattering matrix. It can be estimated with low computational cost in the quasi‐steady limit [53] but for complex geometries, effective high‐resolution numerical solutions for the local problem are a viable option. These computations can be performed using full 3D flow solvers and system identification techniques, either in the time domain [54] or frequency domain [55] with wave decomposition. Such approaches could be valuable for future research in bifurcation modeling.

## 3 Validation

Three well‐documented cases have been selected to validate the proposed formalism and BEATLAB code. The validation begins with a healthy bifurcation, where the normal vessel and bifurcation elements can be tested. Next, a vessel featuring varying degrees of stenosis is analyzed. Finally, a full aorta model is studied. Results are presented graphically (in the time domain), and error quantification is carried out based on the methodology defined by Boileau et al. [56]:

(24) $$\begin{array}{ccc} \varepsilon_{p}^{\mathrm{rms}} & =\sqrt{\frac{1}{n}\sum_{i=1}^{n}{\left(\frac{p_{i}^{m}-p_{i}^{\mathrm{ref}}}{p_{i}^{\mathrm{ref}}}\right)}^{2}}, & \varepsilon_{q}^{\mathrm{rms}}=\sqrt{\frac{1}{n}\sum_{i=1}^{n}{\left(\frac{q_{i}^{m}-q_{i}^{\mathrm{ref}}}{\max\left(q^{\mathrm{ref}}\right)}\right)}^{2}}\\ \varepsilon_{p}^{\max} & =\mathrm{ma}x_{i}\left|\frac{p_{i}^{m}-p_{i}^{\mathrm{ref}}}{p_{i}^{\mathrm{ref}}}\right|, & \varepsilon_{q}^{\max}=\mathrm{ma}x_{i}\left|\frac{q_{i}^{m}-q_{i}^{\mathrm{ref}}}{\max\left(q^{\mathrm{ref}}\right)}\right|\\ \varepsilon_{p}^{\mathrm{sys}} & =\frac{\max\left(p^{m}\right)-\max\left(p^{\mathrm{ref}}\right)}{\max\left(p^{\mathrm{ref}}\right)}, & \varepsilon_{q}^{\mathrm{sys}}=\frac{\max\left(q^{m}\right)-\max\left(q^{\mathrm{ref}}\right)}{\max\left(q^{\mathrm{ref}}\right)}\\ \varepsilon_{p}^{\mathrm{dia}} & =\frac{\min\left(p^{m}\right)-\min\left(p^{\mathrm{ref}}\right)}{\min\left(p^{\mathrm{ref}}\right)}, & \varepsilon_{q}^{\mathrm{dia}}=\frac{\min\left(q^{m}\right)-\min\left(q^{\mathrm{ref}}\right)}{\max\left(q^{\mathrm{ref}}\right)} \end{array}$$

where the superscripts rms,max,sys,dia denote the RMS (Root Mean Square), maximum, systolic, and diastolic errors, respectively. The subscript p,q corresponds to pressure and flow, while the superscripts m,ref refer to model and reference data, respectively. The subscript i indicates the time sample (stamp) $\left(i=1,\ldots ,n\right)$. Reference values are taken from published 3D simulations [45, 57]. For all validation cases, the inlet boundary condition is prescribed using the inlet volume flow data from the references and applying a very high source impedance, set here as $Z_{s}=10^{30}$ [Pas/m^3^].

### 3.1 Case 1: A Healthy Aortic Bifurcation

**FIGURE 5:**
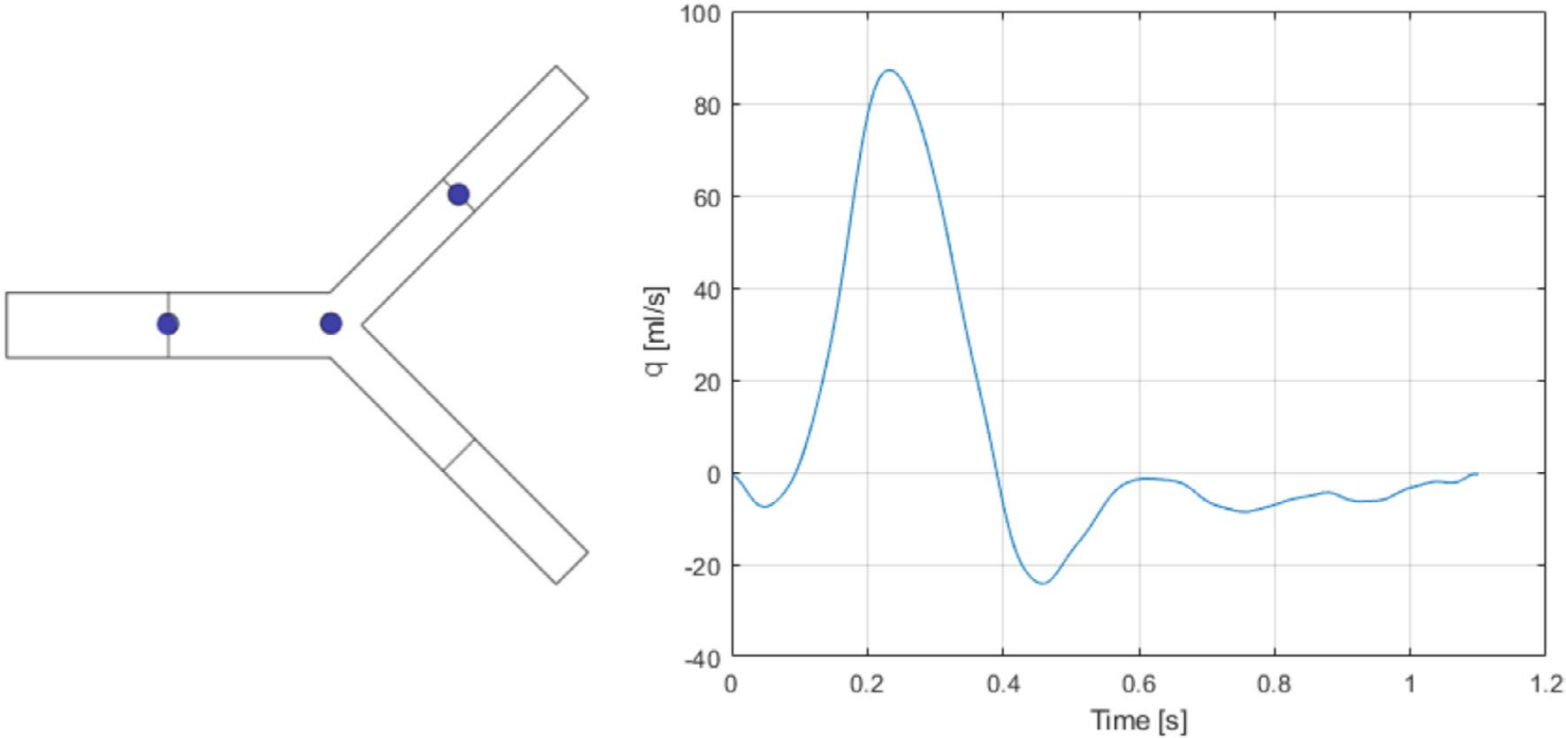
Left: Aortic bifurcation schematic—the dots show mid aorta, junction, and left iliac. Right: Inflow boundary condition.

**FIGURE 6:**
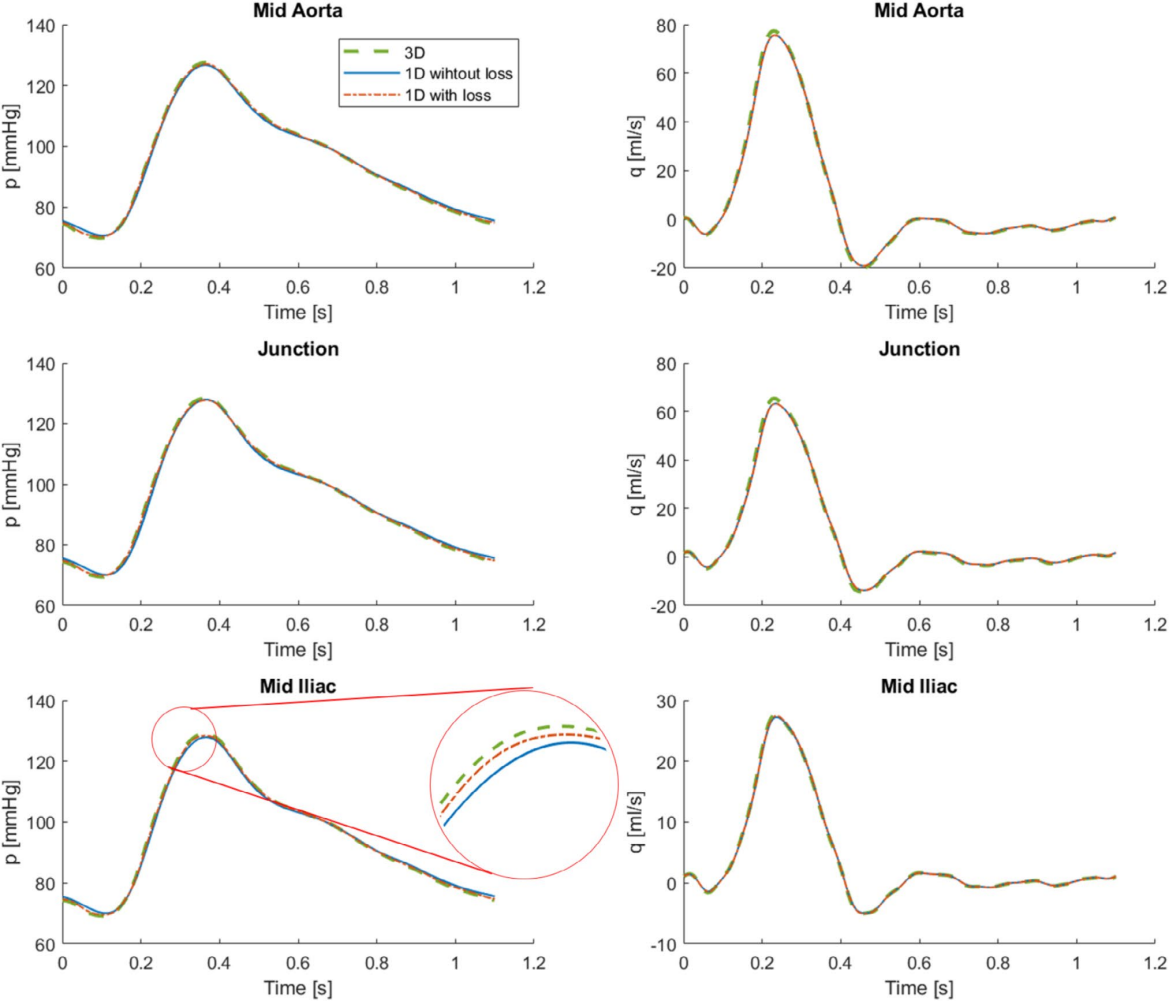
Pressure and flow rate at three locations along the bifurcation, see Figure 5, comparing the 1D model (with and without losses over the bifurcation) with a 3D model [57].

This is one of the benchmark cases from Boileau et al. [56] where results for different 1D codes were compared to reference 3D simulations [57]. It was also utilized by Flores et al. [33] to validate their frequency‐domain code. The geometry is an idealized single bifurcation of the abdominal aorta into the two iliac arteries at a split angle of 51.7°. In the reference papers, the bifurcation is represented using three two‐port elements with uniform properties, connected at an idealized (lossless) node. This case was run first, then the flow losses over the node were included using the procedure outlined in Section 2.4. It is implemented using Equation (19) with $A_{s}=A_{m}$ (the area of the downstream flow branch m), $\sigma_{m}=0$ (No area change in the bifurcation vessels) and $C_{L}=0.1$ [47] symmetrically for both flow paths. Vessel, blood, and boundary (Windkessel parameters) properties are provided in Table 1, while the geometry and inlet volume flow are depicted in Figure 5. Results for pressure and volume flow are obtained at three monitoring positions: the midpoint of the aorta, the bifurcation junction, and the midpoint of one iliac vessel. These results are presented in Figure 6, with quantified errors summarized in Table 2.

**TABLE 1:** Aortic bifurcation. Model parameters from [57].

Properties	Aorta	Iliac
Length	86 mm	85 mm
Radius at diastolic pressure	8.6 mm	6 mm
Average radius	8.9 mm	6.15 mm
Wall thickness	1.032 mm	0.72 mm
Blood density	1060 kg/m^3^	1060 kg/m^3^
Blood viscosity	4 mPa.s	4 mPa.s
Young's modulus	500 kPa	700 kPa
Diastolic pressure	9.1 kPa	9.1 kPa
Outflow pressure	0	0
Windkessel resistance	NA	6.1823 × 10^7^ Pa.s/m^3^
Windkessel compliance	NA	3.6664 × 10^−10^ m^3^/Pa
Windkessel resistance	NA	3.1013 × 10^9^ Pa.s/m^3^

**TABLE 2:** Aortic bifurcation. Error in % compared to 3D model [57].

Error	Mid aorta		Junction		Mid Iliac	
	$p\left[\%\right]$	$q\left[\%\right]$	$p\left[\%\right]$	$q\left[\%\right]$	$p\left[\%\right]$	$q\left[\%\right]$
Without junction loss						
RMS	0.92	1.20	0.93	1.43	0.97	1.94
Max.	1.90	4.48	1.89	4.21	2.03	7.15
Sys.	1.16	−0.20	1.29	0.66	1.42	3.76
Dias.	−1.87	−0.07	−1.86	−0.89	−1.93	−2.26
With junction loss						
RMS	0.29	0.81	0.31	1.1	0.31	0.28
Max.	0.62	2.73	0.58	3.84	0.59	0.95
Sys.	−0.37	−2.4	−0.43	−3.26	−0.39	−0.58
Dias.	0.51	0.79	0.57	0.99	0.54	−0.11

The agreement between the modeled results and the reference 3D simulations is good already for the idealized node conditions; preserving signal dynamics and achieving RMS errors of less than 2% for both pressure and volume flow rate. Most often this is sufficient and a reasonable simplification when modeling healthy vascular systems. However, adding the losses at the bifurcation, the results are improved even further, reducing the RMS error to much less than 1% in most cases. This will be increasingly important for cases with imperfect flow conditions. This outcome serves as validation for the key elements required to construct more complex vascular networks and also the procedure to include flow losses across bifurcations.

### 3.2 Case 2: Stenosis in the Common Carotid Artery (CCA) [45]

**FIGURE 7:**
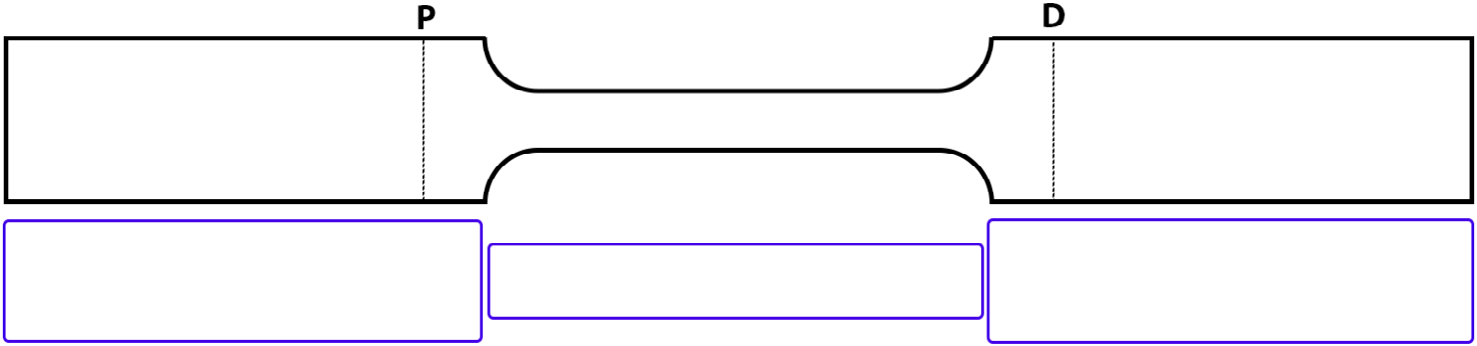
Idealized common carotid with stenosis. As modelled in (Top) 3D [45], (Bottom) 1D model presented here with sudden area changes, see Section 2.3.3.

**FIGURE 8:**
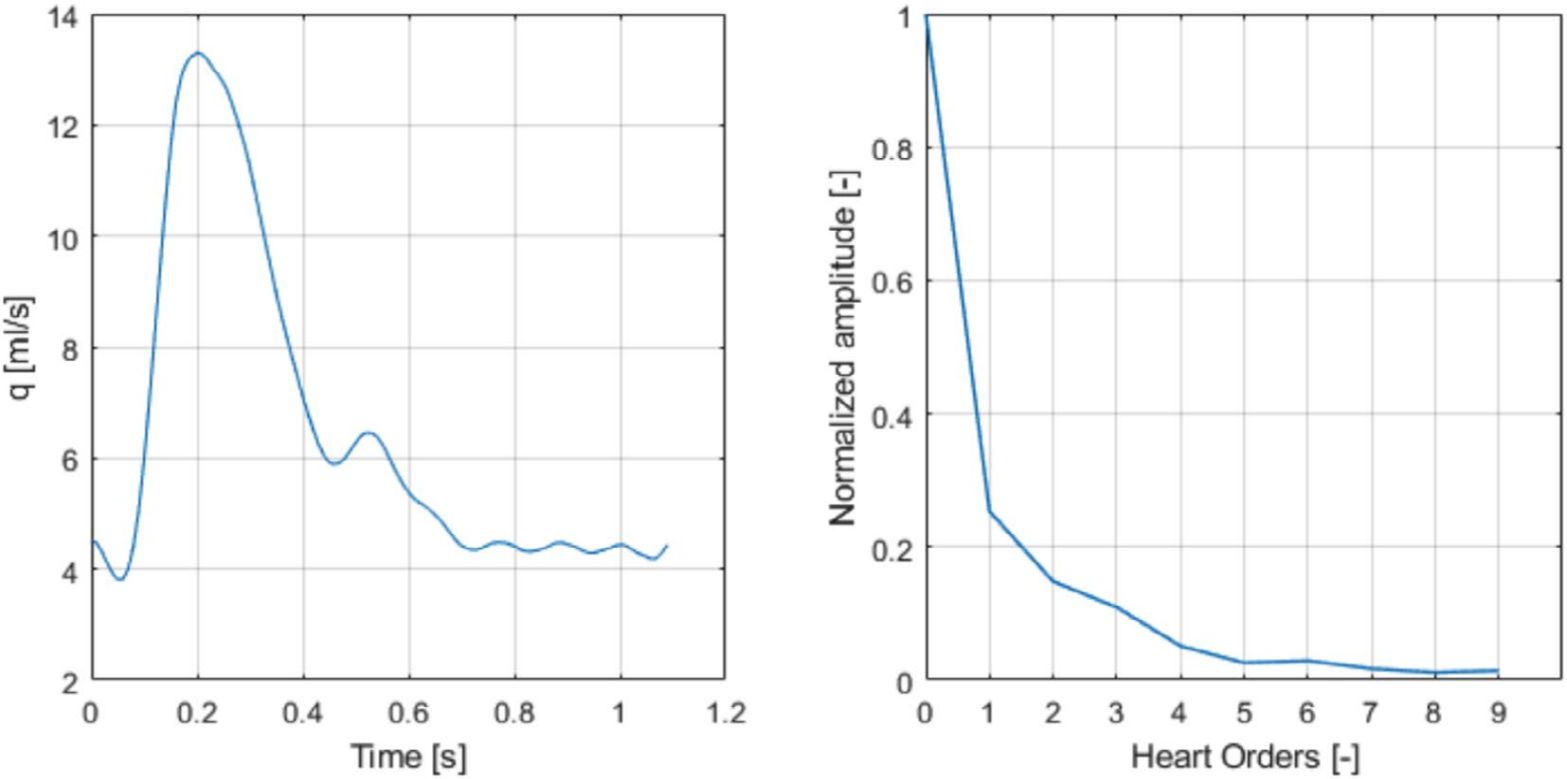
Inflow for common carotid with stenosis, left is the time domain and right is the frequency domain showing the normalized magnitude of each harmonic.

**FIGURE 9:**
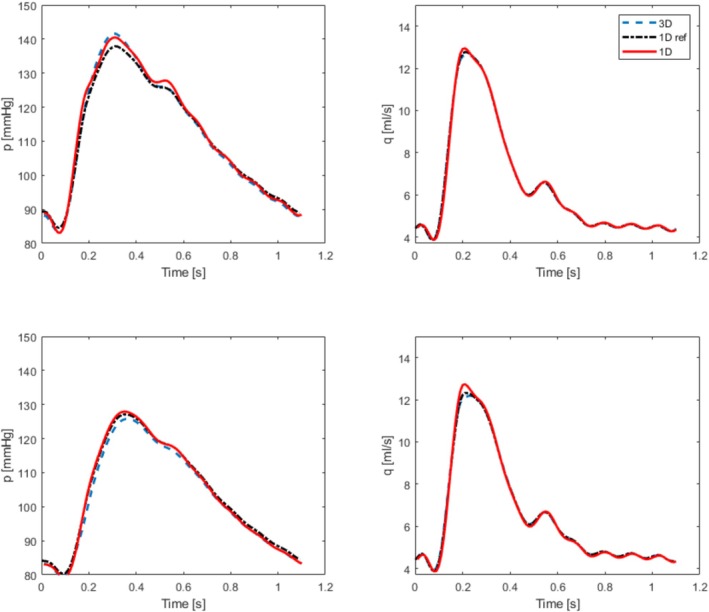
Pressure and volume velocity for the 75% stenosis size with the nonlinear element. The dashed and the dash‐dotted lines are the reference [45] 3D and 1D time domain models, respectively. Row 1 at the Proximal position. Row 2 at the Distal position.

The nonlinear stenosis model proposed in Section 2.3.3 will be validated against the 1D and 3D time domain models of a single stenosis of varying size in an idealized CCA presented by Jin and Alastruey [45]. The relative size of the stenosis is defined as $\sigma_{s}=\left(1-A_{s}/A_{0}\right)$100%, where $A_{s}$ is the minimum cross‐sectional area of the stenosis and $A_{0}$ is the carotid artery cross sectional area at the proximal position. The geometry and model parameters of the CCA are given in Figure 7 and Table 3, respectively, while the inflow condition with the corresponding harmonic content is given in Figure 8. It is clear that for this case, the steady flow (0 Hz) component dominates the flow; the first harmonic is the largest but is only around (1/4) of the 0 Hz component. This implies that the 0 Hz flow component will dominate the flow separation losses, and the proposed simplified nonlinear model can be used (see Equation (19)). Three cases of stenosis, 25%, 50%, and 75%, were simulated using Equation (16), with and without the nonlinear two‐port, using the values $C_{L,\text{in}}=0.52$ and $C_{L,\text{out}}=1$, as proposed in Section 2.3.3. In comparison, the reference 1D time domain model [45] adds the nonlinear effect through a flow speed dependent viscosity. The formula used there is similar to the model proposed here and is also based on a total flow loss coefficient of 1.52. The main difference lies in the distribution of losses, the reference 1D time domain model assumes a uniform distribution along the stenosis section, whereas our model localizes the nonlinear losses at the inlet and outlet. From a fluid mechanics perspective, the localized losses provide a more accurate representation of the actual scenario. The error estimations for all three sizes of the stenosis for the proposed 1D model are given in Table 4. As expected from previous studies [15], the two smaller cases of stenosis compared favorably with the 3D results also without the nonlinear two‐port. However, for the 75% stenosis, the nonlinearity is important and must be included in the model. The pressure and volume flow for the case of 75% stenosis in the proximal and distal positions are presented in Figure 9.

**TABLE 3:** Common carotid. Model parameters from [45].

Properties	Value
Total length	126 mm
Stenosis length	48 mm
Radius at diastolic pressure	3 mm
Average radius	3 mm
Wall thickness	0.3 mm
Blood density	1060 kg/m^3^
Blood viscosity	4 mPa.s
Young's modulus	700 kPa
Diastolic pressure	10.933 kPa
Outflow pressure	0
Windkessel resistance R1	2.4875 × 10^8^ Pa.s/m^3^
Windkessel compliance C	1.7529 × 10^−10^ m^3^/Pa
Windkessel resistance R2	1.8697 × 10^9^ Pa.s/m^3^

**TABLE 4:** Stenosis simulation. Error in % for the proposed 1D model compared to the reference 3D model [45].

Error	Nonlinear model				Linear model			
	$p_{\text{proximal}}$%	$p_{\text{distal}}$%	$q_{\text{proximal}}$%	$q_{\text{distal}}$%	$p_{\text{proximal}}$%	$p_{\text{distal}}$%	$q_{\text{proximal}}$%	$q_{\text{distal}}$%
Stenosis size 25%								
RMS	0.67	0.67	0.32	0.64	0.65	0.67	0.31	0.64
Max.	1.27	1.18	1.06	1.74	1.19	1.19	1.03	1.75
Sys.	0.94	1.02	−0.61	1.29	0.88	1.02	−0.61	1.29
Dias.	−0.89	−0.99	−0.09	−0.63	0.90	−0.99	−0.09	−0.64
Stenosis size 50%								
RMS	0.59	0.82	0.35	0.61	0.42	0.84	0.31	0.62
Max.	1.31	1.81	1.31	1.76	0.89	1.87	1.13	1.90
Sys.	0.73	1.31	−0.31	1.6	0.24	1.32	−0.27	1.65
Dias.	−0.76	−0.83	−0.26	−0.75	−0.86	−0.84	−0.31	−0.78
Stenosis size 75%								
RMS	1.17	1.4	0.87	1.25	2.63	1.61	0.90	1.48
Max.	4.08	4.33	3.29	5.45	7.26	5.35	3.48	6.43
Sys.	−0.79	1.69	2.44	4.49	−5.65	1.73	2.86	5.08
Dias.	−0.41	−0.41	−0.19	−0.19	−1.70	−0.70	−0.76	−1.19

In conclusion, the model with sudden area changes used here effectively captured the shape of the pulse wave curves. Furthermore, the proposed new nonlinear frequency domain model for flow separation losses performed at least as well as the 1D time domain model in Ref. [45]. In this test case, the new nonlinear model converged very quickly, requiring <5 iterations. The solution time is 0.2 s on a standard office laptop; two orders of magnitude (100 times) shorter compared to the reference 1D time domain model reported by Jin et al. [45].

### 3.3 Case 3: Full Aorta Model

**FIGURE 10:**
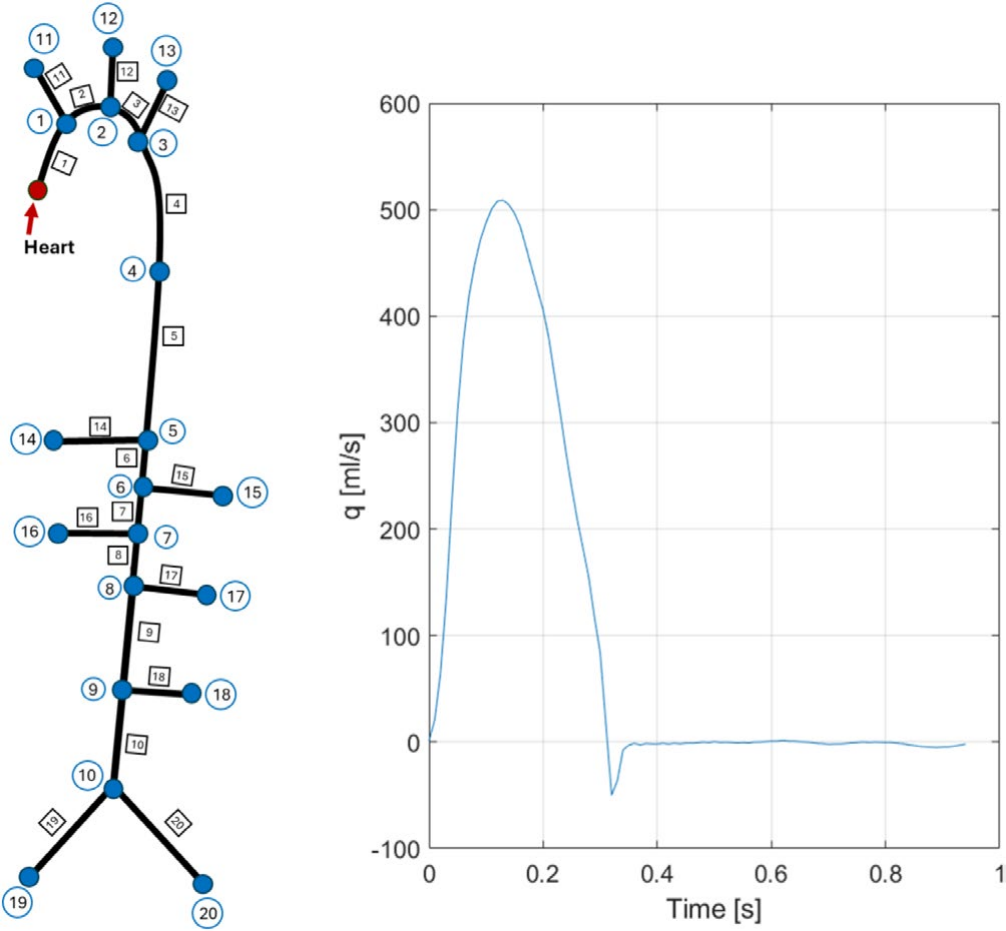
Left: Aorta network representation. Right: Inflow boundary condition.

The third case is a “full aorta,” including its main branches and bifurcation [57]. It represents a healthy aorta without any abnormalities, implying that no nonlinear effects will be included. The 1D model comprises 20 arterial two‐port elements with constant properties and 21 nodes, 10 of which are Windkessel elements and one representing a heart element (constant volume flow source). The nodes between elements were assumed to be lossless. The network schematic and inlet flow are presented in Figure 10, with model parameters tabulated in Table 5.

**TABLE 5:** Full aorta model parameters.

Arterial segment	Length (cm)	*R*_*d*_ (mm)	*R*_0_ (mm)	E (kPa)	R_1_ × 10^7^ (Pa.s.m.^−3^)	R^2^ × 10^8^ (Pa.s.m.^−3^)	C × 10^−10^ (Pa.s.m.^−3^)
1. Ao I	7.04	14.55	14.59	372.2	—	—	—
2. Ao II	0.8	13.8	13.8	384.2	—	—	—
3. Ao III	0.9	13.60	13.65	387.6	—	—	—
4. Ao IV	6.47	12.9	12.9	400.0	—	—	—
5. Ao V	15.2	11.1	11.1	437.8	—	—	—
6. Ao VI	1.8	9.8	9.8	471.8	—	—	—
7. Ao VII	0.7	9.66	9.66	475.9	—	—	—
8. Ao VIII	0.7	9.585	9.6	478.1	—	—	—
9. Ao IX	4.3	9.31	9.31	486.5	—	—	—
10. Ao X	4.3	8.88	8.88	502.0	—	—	—
11. Brach.	3.4	6.35	6.35	612.0	5.1918	10.6080	8.6974
12. L com. carotid	3.4	3.6	3.6	860.4	19.1515	52.2129	1.7670
13. L subclavian	3.4	4.8	4.8	724.0	9.8820	13.0183	7.0871
14. Celiac	3.2	4.45	4.45	757.6	11.7617	7.5726	12.1836
15. Sup. mesenteric	6	3.75	3.75	839.6	17.4352	5.5097	16.7453
16. R renal	3.2	2.8	2.8	1000.4	34.1378	5.3949	17.1017
17. L renal	3.2	2.8	2.8	1000.4	34.1378	5.3949	17.1017
18. Inf.mesenteric	5	2.0	2.0	1224.2	74.0167	46.2252	1.9959
19. R com. iliac	8.5	6.0	6.0	633.3	5.9149	10.1737	9.0686
20. L com. iliac	8.5	6.0	6.0	633.3	5.9149	10.1737	9.0686

*Note:* Parameters are from Xiao et al. [57] where values for diastolic radii at the inlet *R* 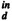 and diastolic radii at the outlet *R* 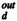, are given. The computation of the results according to [33] assumed an averaged diastolic radius, $R_{d}=\left(R_{d}^{\text{in}}+R_{d}^{\text{out}}\right)/2$, the wall thickness h is set to 10% of $R_{d}$ and the elastic moduli were calculated using $E=\left(3\rho_{0}c_{0}^{2}R_{d}\right)/2h.$ Blood density and viscosity have the same values as in Case 1 and 2.

This case provides the opportunity to evaluate how the 1D simulation replicates a more complex network featuring large elastic vessels that are also curved and tapered. In line with common practice [56], curved vessels are represented as straight elements of the centerline lengths of the vessels. Simulations were initially performed with and without tapering. The effect was negligible; in the results presented, the tapered elements are replaced by constant diameter vessels having the mean radius.

**FIGURE 11:**
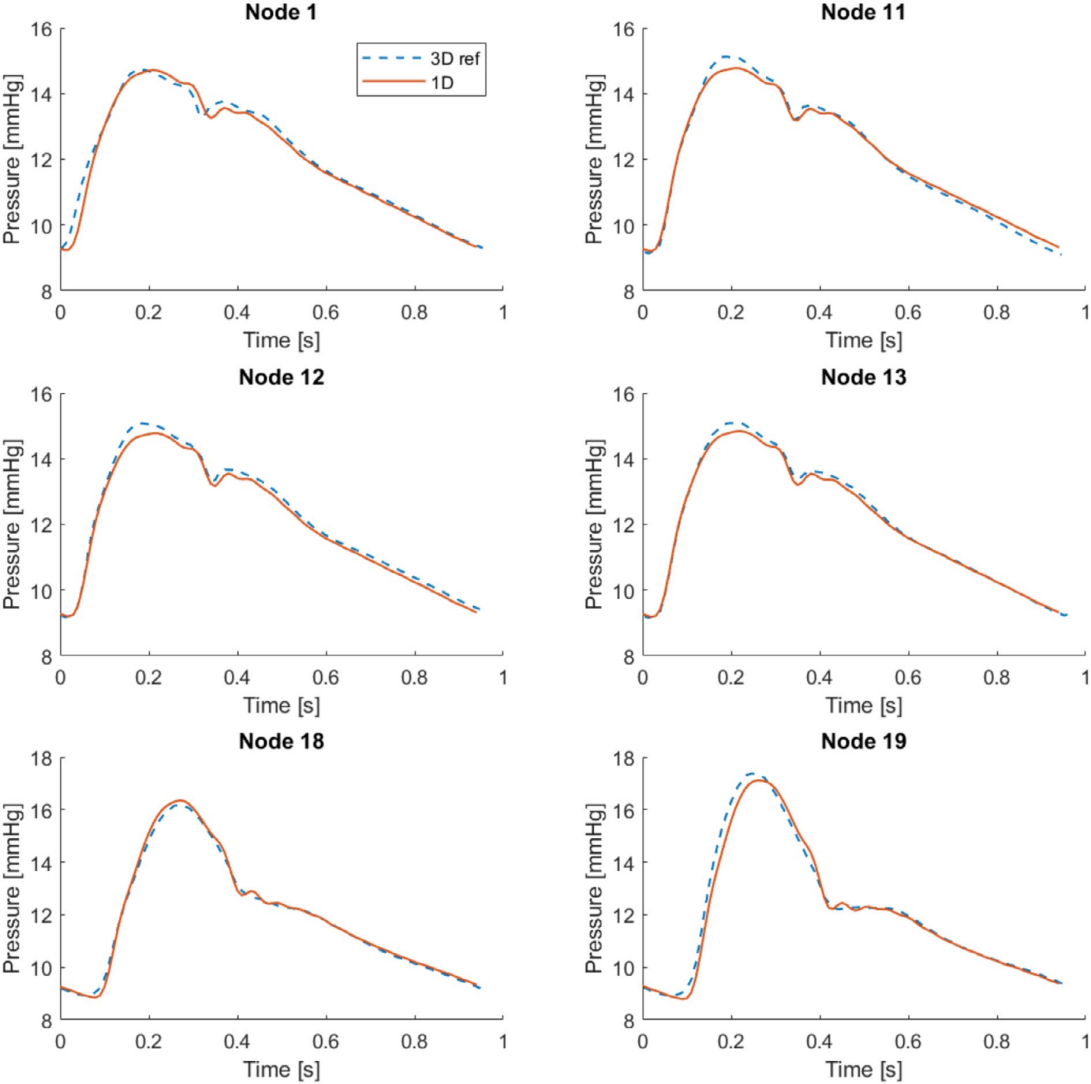
Full aorta model results. Reference 3D results from Xiao et al. [57] compared to the 1D model presented here. Node numbering can be found in Figure 10.

Pressure data at six different nodes along the network is compared with 3D results [57], in Figure 11. The error estimations, calculated using Equation (24), are summarized in Table 6. The results show excellent agreement across all nodes with the 3D results, providing accurate estimations of the systolic, diastolic, and slope of the pressure curve. Furthermore, minor details and inflection points are well captured.

**TABLE 6:** Aorta tree simulation. Error in % compared to 3D model [57] at six nodes.

Error	$p_{1}$%	*p*_11_%	*p*_12_%	*p*_13_%	*p*_18_%	*p*_19_%
RMS	2.02	1.13	1.28	0.87	1.1	2.38
Max.	8.54	2.78	2.67	2.03	3.69	6.27
Sys.	−0.01	−2.31	−1.95	−1.65	1.13	−1.48
Dias.	−1.65	0.55	0.12	0.13	−0.79	−1.36

## 4 Discussion

It has been shown that the proposed low‐order formalism reproduces the reference cases with as good as or better precision than existing low‐order codes. In addition, the proposed frequency domain method is computationally much faster than time‐domain methods. Losses or active sources in the nodes in between vessel elements are an integral part of the solution and do not add computational cost. The introduction of local nonlinearities in the frequency domain solution proved successful, overcoming one of the most common drawbacks of Fourier methods. This opens avenues for effective creation of virtual patient cohorts, training data for machine learning models, or even solving digital twin optimization problems.

However, no model—regardless of fidelity—is better than the data fed to it. In the application examples presented, all material, geometry, and boundary condition data were known. In clinical applications, or even when creating new virtual cohorts, many of these parameters are unavailable or estimated via indirect methods [58]. For vessel geometry, automated imaging and segmentation techniques are rapidly advancing. Imaging methods can also provide local approximations of wave propagation speed, while basic markers such as blood pressure and PWV offer indirect estimations of peripheral resistance and vessel wall material properties when available. However, such measurements often require costly hardware, sophisticated software, and trained personnel, and they still involve significant uncertainty. Setting realistic parameter variations for different target cohorts (or optimization routines) is therefore often one of the main challenges.

A useful approach to augment sparse datasets is to start with “best practice” generic parameter variations, establishing a parameter space. Physiologically unrealistic parameter combinations can then be disqualified using a limited but specific dataset for the target cohort. Ideally, improved conditional parameter variations can be established before producing large cohorts. An example of the first stage of this strategy is the pulse wave database by Charlton et al. [9], which provides nominal parameter values and their standard deviations as a function of age based on a meta‐study of individual parameter variations for healthy individuals. Once the full parameter space is established, in vivo population data, such as that from McEniery et al. [59] (including brachial and aortic blood pressure, return time, etc.), can be used to retain only physiologically realistic combinations for the specific target cohort.

For anomalies, useful reference data is even scarcer. Not only do the geometry and material composition of the stenosis or aneurysm vary, but boundary conditions—such as vasodilation downstream of a stenosis to compensate for circulatory loss—also often change. Understanding these types of co‐variations remains an area of significant research interest. Another practically important area involves integrating bio‐sensors into the modeling framework. Promising noninvasive sensors such as blood pressure cuffs, PhotoPlethysmoGraphy (PPG), or radar sensors measure proxies for the actual arterial quantities of interest.

## Author Contributions

**Mikael Karlsson:** conceptualization, methodology, investigation, writing – original draft, review and editing, visualization, project administration, funding acquisition. **Mina Nashed:** methodology, software, validation, visualization, writing – original draft, review and editing. **Tamer Elnady:** conceptualization, methodology, software, funding acquisition. **Mats Åbom:** conceptualization, methodology, software, validation, writing – original draft, review and editing.

## Ethics Statement

The authors have nothing to report.

## Conflicts of Interest

Mikael Karlsson, Mats Åbom, and Tamer Elnady have interests in Beat Vascular Health AB, providing the software BEATLAB. The other author declares no conflicts of interest.

## Figures and Tables

**FIGURE B1:**
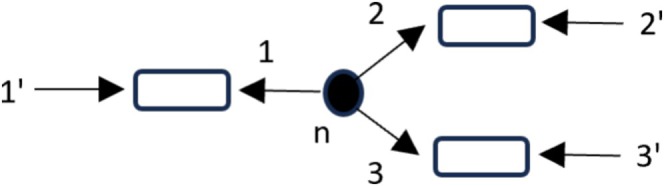
Schematic of three‐port constructed from three two‐port elements. Compare to definition of nodes and elements in Figure 1.

## Data Availability

The data that support the findings of this study are available from the corresponding author upon reasonable request.

## Appendix A Transformation From Transfer Matrix to Scattering Matrix

The backward transfer matrix between the outlet (2) and inlet (1); see Figure 1, is given by

(A1)
$$\left[\begin{array}{c} {\hat{p}}_{1}\\ {\hat{q}}_{1} \end{array}\right]=\left[\begin{array}{cc} A & B\\ C & D \end{array}\right]\left[\begin{array}{c} {\hat{p}}_{2}\\ {\hat{q}}_{2} \end{array}\right]$$

Note that the positive direction for the volume flow is from 1 to 2, see Figure 1. Using the relation (see Equation (4)) between pressure and volume velocity for waves propagating in the ± direction, we get

(A2)
$$\left[\begin{array}{c} {\hat{p}}_{1+}+{\hat{p}}_{1-}\\ {\hat{p}}_{1+}/Z_{1}-{\hat{p}}_{1-}/Z_{1} \end{array}\right]=\left[\begin{array}{cc} A & B\\ C & D \end{array}\right]\left[\begin{array}{c} {\hat{p}}_{2-}+{\hat{p}}_{2+}\\ {\hat{p}}_{2-}/Z_{2}-{\hat{p}}_{2+}/Z_{2} \end{array}\right]$$

The wave impedance Z is singular at 0 Hz, which can be handled by using a small but nonzero value for the frequency. Rearranging with outgoing wave amplitudes on the left‐hand side and incident on the right‐hand side gives

(A3)
$$\left[\begin{array}{cc} 1 & -A-B/Z_{2}\\ -1/Z_{1} & -C-D/Z_{2} \end{array}\right]\left[\begin{array}{c} {\hat{p}}_{1-}\\ {\hat{p}}_{2-} \end{array}\right]=\left[\begin{array}{cc} -1 & A-B/Z_{2}\\ -1/Z_{1} & C-D/Z_{2} \end{array}\right]\left[\begin{array}{c} {\hat{p}}_{1+}\\ {\hat{p}}_{2+} \end{array}\right]$$

Based on the definition (Equation (2)) of the scattering matrix, it follows that

(A4)
$$S={\left[\begin{array}{cc} 1 & -A-B/Z_{2}\\ -1/Z_{1} & -C-D/Z_{2} \end{array}\right]}^{-1}\left[\begin{array}{cc} -1 & A-B/Z_{2}\\ -1/Z_{1} & C-D/Z_{2} \end{array}\right]$$

## Appendix B Three‐Port Node Derivation

Derive a three‐port scattering matrix with interfaces at the primed cross‐sections including the three two‐ports connected to the node scattering matrix $S_{n}$ (Figure B1).

Assumptions:

- Three‐port node that can be ideal.
- No internal source (can be relaxed).
- Node connected to three two‐port elements.

From the three two‐port element scattering matrices $S_{i}$, see Equation (2), one can solve for the transfer matrix from cross‐section *i*  to *i* (i=1,2,3):

(B5)
$$\left\{\begin{array}{c} {\hat{p}}_{i-}=S_{i,11}{\hat{p}}_{i+}+S_{i,12}{\hat{p}}_{i^{\prime }+}\\ {\hat{p}}_{i^{\prime }-}=S_{i,21}{\hat{p}}_{i+}+S_{i,22}{\hat{p}}_{i^{\prime }+} \end{array}\right.$$

rearranging yields,

(B6)
$$\left[\begin{array}{c} {\hat{p}}_{i+}\\ {\hat{p}}_{i-} \end{array}\right]=T_{i}\left[\begin{array}{c} {\hat{p}}_{i^{\prime }+}\\ {\hat{p}}_{i^{\prime }-} \end{array}\right]$$

with

(B7)
$$T_{i}=\left[\begin{array}{cc} \left(-\frac{S_{i,22}}{S_{i,21}}\right) & \left(\frac{1}{S_{i,21}}\right)\\ \left(S_{i,12}-\frac{S_{i,11}S_{i,22}}{S_{i,21}}\right) & \left(\frac{S_{i,11}}{S_{i,21}}\right) \end{array}\right]$$

Combine Equation (B6) with the definition of the node scattering matrix $S_{n}$, see Equation (23), one can now derive the three‐port node with interfaces at the primed cross‐sections:

(B8)
$$\left[\begin{array}{c} T_{1,11}{\hat{p}}_{1^{\prime }+}\\ T_{2,11}{\hat{p}}_{2^{\prime }+}\\ T_{3,11}{\hat{p}}_{3^{\prime }+} \end{array}\right]+\left[\begin{array}{c} T_{1,12}{\hat{p}}_{1^{\prime }-}\\ T_{2,12}{\hat{p}}_{2^{\prime }-}\\ T_{3,12}{\hat{p}}_{3^{\prime }-} \end{array}\right]=S_{n}\left(\left[\begin{array}{c} T_{1,21}{\hat{p}}_{1^{\prime }+}\\ T_{2,21}{\hat{p}}_{2^{\prime }+}\\ T_{3,21}{\hat{p}}_{3^{\prime }+} \end{array}\right]+\left[\begin{array}{c} T_{1,22}{\hat{p}}_{1^{\prime }-}\\ T_{2,22}{\hat{p}}_{2^{\prime }-}\\ T_{3,22}{\hat{p}}_{3^{\prime }-} \end{array}\right]\right)$$

Note that the outward direction for a node is defined opposite to the two ports, hence the change in sign for the incident waves. Introducing a more compact matrix and vector notation:

(B9)
$$T_{11}{\hat{p}}_{+}^{\prime }+T_{12}{\hat{p}}_{-}^{\prime }=S_{n}\left(T_{21}{\hat{p}}_{+}^{\prime }+T_{22}{\hat{p}}_{-}^{\prime }\right)$$

(B10)
$$\left(T_{12}-S_{n}T_{22}\right){\hat{p}}_{-}^{\prime }=\left(S_{n}T_{21}-T_{11}\right){\hat{p}}_{+}^{\prime }$$

where $T_{\mathrm{ij}}=\left[\begin{array}{ccc} T_{1,\mathrm{ij}} & 0 & 0\\ 0 & T_{2,\mathrm{ij}} & 0\\ 0 & 0 & T_{3,\mathrm{ij}} \end{array}\right]$ is a diagonal matrix with i,j=1,2,3. The new scattering matrix with interfaces at the primed cross‐sections is:

(B11)
$$S\prime_{n}={\left(T_{12}-S_{n}T_{22}\right)}^{-1}\left(S_{n}T_{21}-T_{11}\right)$$
